# Integrative epigenetic and transcriptomic profiling of whole blood and fibroblasts in Hao-Fountain syndrome

**DOI:** 10.3389/fcell.2026.1782599

**Published:** 2026-02-20

**Authors:** Liselot van der Laan, Rob Zwart, Andrea Venema, Adri N. Mul, Martin A. Haagmans, Bart Hulsbosch, David Dyment, Irene Valenzuela, Pilar Caro, Sebastian Sailer, Christian P. Schaaf, Bekim Sadikovic, Marcel M. A. M. Mannens, Mieke M. van Haelst, Manasa Kalya Purushothama, Peter Henneman

**Affiliations:** 1 Department of Human Genetics, Amsterdam UMC, Amsterdam, Netherlands; 2 Amsterdam Reproduction & Development, Amsterdam University Medical Centers, University of Amsterdam, Amsterdam, Netherlands; 3 Children’s Hospital of Eastern Ontario Research Institute, Ottawa, ON, Canada; 4 Àrea de Genètica Clínica i Malalties Minoritàries, Hospital Vall d'Hebron, Barcelona, Spain; 5 Institute of Human Genetics, Heidelberg University, Heidelberg, Germany; 6 London Health Science Centre, Verspeeten Clinical Genome Centre, London, ON, Canada; 7 Department of Pathology and Laboratory Medicine, Western University, London, ON, Canada; 8 Emma Center for Personalized Medicine, Amsterdam UMC, Amsterdam, Netherlands

**Keywords:** BCOR, DNA methylation, eQTM, gene expression, H2AK119ub1, Hao–Fountain syndrome, hox genes, PRC1.1

## Abstract

**Background:**

Hao–Fountain syndrome (HAFOUS) is a rare autosomal dominant neurodevelopmental disorder caused by pathogenic *USP7* variants. A diagnostic blood DNA methylation episignature has been established, yet the broader regulatory consequences of *USP7* haploinsufficiency and their tissue specificity remain incompletely characterized.

**Methods:**

We performed genome-wide DNA methylation profiling, RNA sequencing, and cis expression quantitative trait methylation (eQTM) analysis in whole blood (n = 9) and patient-derived skin fibroblasts (n = 4). Differential methylation was assessed and methylation–expression coupling within ±250 kb of each DMR. DMRs were further interpreted using BCOR, H2AK119ub1, and H3K27me3 ChIP-Rx datasets from neural models.

**Results:**

Blood reproduced the established *USP7* hypermethylation episignature and yielded 17 significant DMRs, accompanied by modest numbers of differentially expressed genes and eQTMs. Fibroblasts displayed internally coherent regulatory patterns, including 2,143 nominal DMRs, 310 differentially expressed genes, and 559 significant eQTMs. Convergent methylation–expression changes prominently involved the HOXB cluster (HOXB3, HOXB5, HOXB6). Both blood- and fibroblast-derived DMRs showed significant enrichment for BCOR- and H2AK119ub1-marked regions, consistent with disruption of non-canonical PRC1.1–associated chromatin. Cross-tissue comparison revealed limited overlap, supporting marked tissue specificity in methylation–expression relationships.

**Conclusion:**

*USP7* haploinsufficiency is associated with a restricted set of regulatory loci enriched within PRC1-associated chromatin domains. Fibroblasts revealed coherent methylation and expression changes at developmental genes, whereas blood captured the diagnostic episignature and a smaller set of downstream regulatory alterations. Together, this dual-tissue integrative analysis refines the molecular consequences of reduced *USP7* dosage and provides a framework for future mechanistic studies in disease-relevant cellular models.

## Introduction

Hao–Fountain syndrome (HAFOUS) is a rare autosomal dominant neurodevelopmental disorder caused by pathogenic variants or deletions involving *USP7* (OMIM #602519). Affected patients typically present with developmental delay, intellectual disability, behavioral abnormalities, autism spectrum disorder, seizures, hypogonadism, and mild dysmorphic facial features (Fountain et al., 2019; Hao et al., 2015; Wimmer et al., 2024). USP7, located on chromosome 16p13.2, encodes a deubiquitinating enzyme with broad regulatory roles in cellular homeostasis. Canonically, *USP7* stabilizes MDM2 and thereby modulates P53 turnover, influencing DNA repair, transcriptional responses, and genome integrity (Li et al., 2002; Schaefer and Morgan, 2011; Nicholson and Suresh Kumar, 2011; van der Laan et al., 2024).

Recent work has expanded this functional repertoire by demonstrating that USP7 acts as a dosage-sensitive regulator of Polycomb-mediated chromatin repression. Loss-of-function experiments revealed that BCOR–ncPRC1.1, rather than ncPRC1.6, is the principal Polycomb effector responsive to USP7 dosage during neuronal differentiation, with downstream effects on deposition of H2AK119ub1 (Wolf van der Meer et al., 2025). USP7 additionally interacts with other ubiquitin-regulated chromatin pathways, including substrates such as TRIM27, underscoring its broader role in shaping chromatin-associated ubiquitin states (Nasreen et al., 2025; Mircea and Semrau, 2021). Together, these findings provide a mechanistic basis for how germline USP7 haploinsufficiency may disrupt Polycomb-regulated developmental programs relevant to HAFOUS.

DNA methylation episignatures have emerged as robust diagnostic biomarkers across a spectrum of Mendelian neurodevelopmental syndromes (Sadikovic et al., 2019; Sadikovic et al., 2020; Aref-Eshghi et al., 2021). We recently established and validated a blood-derived *USP7* episignature for HAFOUS (van der Laan et al., 2023). However, blood-based methylation profiles may not fully capture regulatory alterations occurring in developmentally relevant tissues (Dajani et al., 2013; Cuvertino et al., 2025; Awamleh et al., 2024). Fibroblasts, in contrast, provide a homogeneous, lineage-consistent, and experimentally tractable model system that can reveal stable regulatory consequences of germline variation. They have been successfully used to study molecular mechanisms in disorders involving chromatin and transcriptional regulators, including Rett, Kabuki, and Koolen-de Vries syndrome (Dajani et al., 2013; Cuvertino et al., 2025; Awamleh et al., 2024). These studies demonstrate that patient-derived skin fibroblasts can reveal stable, cell-intrinsic epigenetic and transcriptional alterations, and in some settings may capture regulatory changes that are less apparent in heterogeneous peripheral blood (Alders et al., 2014).

Most cis-eQTMs occur within several hundred kilobases of transcription start sites, capturing regulatory interactions involving promoters, enhancers, and broader chromatin territories (Hannon et al., 2018; Bonder et al., 2017). Furthermore, publicly available chromatin profiling data from *USP7*- haploinsufficient neural models provide an opportunity to contextualize patient-derived methylation changes relative to Polycomb-associated genomic regions enriched for BCOR, H2AK119ub1, and H3K27me3 (Wolf van der Meer et al., 2025).

Here, we performed a multi-omics analysis of whole blood (n = 9) and patient-derived fibroblasts (n = 4) with HAFOUS, integrating genome-wide DNA methylation, transcriptomic profiling, and eQTM mapping. Our objectives were to (i) identify differentially methylated regions (DMRs) in each tissue, (ii) characterize transcriptomic alterations, (iii) determine cis-regulatory methylation–expression relationships, (iv) compare blood- and fibroblast-derived signatures to distinguish shared versus tissue-specific regulatory changes, and (v) evaluate whether HAFOUS-associated DMRs preferentially localize to Polycomb-enriched chromatin domains implicated in *USP7* dysfunction. Through this integrative approach, we aim to elucidate key regulatory pathways disrupted in HAFOUS and advance understanding of the molecular mechanisms underlying *USP7* haploinsufficiency.

## Methods

### Subjects and study cohort

The study included two sample sets: (i) 9 whole blood from patients with *USP7* variants and unaffected controls, and (ii) 4 primary skin fibroblasts from patients with pathogenic *USP7* variants or deletions and matched healthy controls.

Blood cohort: Nine patients with (likely) pathogenic *USP7* variants and four unaffected controls were included in the DNA methylation study. All nine cases displayed the characteristic *USP7*/HAFOUS methylation episignature (van der Laan et al., 2023) including six patients who had been part of our previously published HAFOUS episignature cohort (van der Laan et al., 2023). High-quality EPIC array data were obtained for all participants; these 13 samples formed the blood whole cohort (n = 9 cases vs. 4 controls), used for DMP and DMR identification.

RNA sequencing was performed on a subset of the blood from the whole cohort consisting of six *USP7* cases and the same four controls. Two case samples failed RNA-seq quality control and were removed, yielding a final set of four *USP7* cases plus four controls with matched methylation and gene expression data. This set is referred to throughout the manuscript as the blood integrative sub-cohort (n = 4 cases vs. 4 controls), used for differential expression and eQTM-analyses. Quality control procedures and specific filtering steps for methylation and RNA-seq are described in later sections.

Fibroblast cohort: Primary skin fibroblasts were collected from four patients with (likely) pathogenic *USP7* variants or microdeletions encompassing the *USP7* locus. Variant classification followed ACMG/AMP and HGVS guidelines (Richards et al., 2015; den Dunnen et al., 2016). Four healthy patients without reported genetic diseases served as fibroblast controls. All eight fibroblast samples passed methylation and RNA-seq quality control and were included in downstream analyses.

### Cell culture

Human skin fibroblasts were maintained in Dulbecco’s Modified Eagle Medium (DMEM) supplemented with 10% fetal calf serum (FCS), 4.5 g/L glucose, 100 U/mL penicillin, 100 μg/mL streptomycin, 20 mM L-glutamine, and 1% MEM Non-Essential Amino Acids (NEAA; Life Technologies). Cultures were kept at 37 °C in a humidified 5% CO_2_ incubator and monitored microscopically for morphology and confluency. Upon reaching ∼80% confluence, cells were passaged using trypsin and either reseeded or processed for downstream experiments.

### DNA extraction, sequencing, and variant validation

Genomic DNA was extracted from whole blood using the Maxwell RSC Whole Blood DNA Kit (AS1520, Promega) and from cultured fibroblasts using the Maxwell RSC Cultured Cells DNA Kit (AS1620, Promega), following the manufacturer’s protocols. Total RNA was treated with RNase-Free DNase I (Thermo Fisher Scientific) to remove genomic DNA contamination, and RNA quantity and integrity were assessed using a NanoDrop spectrophotometer and an Agilent 2,100 Bioanalyzer.

### Blood variant confirmation

(Likely) pathogenic *USP7* variants in the blood whole cohort had been previously established through clinical exome sequencing and/or chromosomal microarray analysis in ISO 15189–accredited diagnostic laboratories. As these variants had undergone prior clinical confirmation, no additional experimental validation was performed in this study.

### Fibroblast variant validation

(Likely) pathogenic *USP7* variants or *USP7*-encompassing microdeletions had been previously identified in accredited diagnostic laboratories. For fibroblast cases 1 and 3, sequence variants were reconfirmed in this study by PCR amplification (∼300 bp amplicons designed with Primer3), followed by agarose gel electrophoresis and Sanger sequencing. Resulting chromatograms were inspected and aligned using IGV v2.11. For fibroblast cases 2 and 4, which carried larger deletions, we performed PCR-free adaptive long-read sequencing using the Oxford Nanopore Ligation Sequencing Kit V14 (SQK-LSK114) on MinION R10.4 flow cells, targeting chr16:4,750,000–9,925,000 (hg19) via adaptive sampling. Read alignments and breakpoints were examined in IGV v2.11.

### DNA methylation profiling

For whole blood and fibroblast samples, 500 ng of genomic DNA was bisulfite-converted using the EZ DNA Methylation Kit (Zymo Research) and processed on the Illumina Infinium MethylationEPIC BeadChip (>850,000 CpG sites) (van Iterson et al., 2014). Raw IDAT files were imported into R (version 4.2.0) using the *minfi* package (version 3.20) (Aryee et al., 2014).

Initial sample-level quality control was performed with *MethylAid* (version 1.30.0) (van Iterson et al., 2014), assessing bisulfite conversion efficiency, probe detection p-values, probe intensity distributions, and outlier status. Functional normalization was applied using preprocessFunnorm (Fortin et al., 2014). Both β-values and M-values were computed for downstream analyses. Probe-level filtering followed standard EPIC array exclusion criteria, removing: probes containing common SNPs (MAF >0.01 at the CpG or extension base), cross-reactive or multi-mapping probes, probes mapping to sex chromosomes, probes with missing or low-quality signal.

### Differential methylation analysis

For the blood whole cohort, cell-type proportions (CD8^+^ T cells, CD4^+^ T cells, B cells, monocytes, neutrophils, and NK cells) were estimated using *estimateCellCounts* function (Koestler et al., 2013) of *minfi* (Aryee et al., 2014) and included as covariates alongside age and sex. For fibroblasts, age at biopsy and sex were used as covariates. Participant characteristics for both cohorts are provided in Supplementary Tables 1a, 1b. Differential methylation analyses were performed separately for blood and fibroblasts using these models.

### Differentially methylated positions

DMPs were identified using limma: lmFit (limma version 3.62.2), incorporating covariates appropriate for each tissue. *For blood:* age, sex, and estimated cell-type proportions (CD8^+^ T cells, CD4^+^ T cells, B cells, monocytes, neutrophils, and NK cells). *For fibroblasts:* age and sex.

Variance estimates were stabilized using empirical Bayes moderation. Multiple testing correction used the Benjamini–Hochberg procedure, and CpGs were considered significant at FDR <0.05, with an absolute mean β-difference cutoff applied as specified. For analyses where no CpGs reached FDR significance (e.g., fibroblasts), nominal p-values were retained to support exploratory and region-level analyses.

### Differentially methylated regions (DMRs)

Two complementary region-calling approaches were used.

Blood cohort: DMRs were identified using DMRcate (v2.10.0) (Peters et al., 2015) with parameters consistent with those used in the EpiSign pipeline. Regions were defined as clusters of ≥5 consecutive DMPs within a 1 kb window and considered significant at FDR <0.05.

Fibroblast cohort: DMRcate (Peters et al., 2015) did not detect regions passing FDR significance, consistent with low power in small-N rare disease cohorts (Peters et al., 2015; Pidsley et al., 2016; Zhou et al., 2017). Therefore, region-level analysis was performed using bumphunter (*minfi*) (Aryee et al., 2014), requiring ≥3 consecutive DMPs, a bump cut-off of 0.15, and 100 bootstraps. Nominally significant regions (P < 0.05) were retained for exploratory integrative analyses, acknowledging that they do not meet family-wise-error-rate (FWER)significance.

### RNA sequencing and transcriptome analysis

#### Sample preparation

Whole blood was collected in PAXgene Blood RNA tubes (PreAnalytiX) and stored at −80 °C prior to extraction. Skin fibroblasts were harvested, and total RNA was isolated using the QIAsymphony system (Qiagen). RNA integrity and concentration were assessed using the Agilent TapeStation, according the manufacturer’s instructions.

#### Library preparation and sequencing

Library preparation was performed at the Centre for Genomics Facilities (CFG) under ISO 15189–certified conditions. Polyadenylated RNA was enriched using the KAPA mRNA HyperPrep Kit (Roche) and converted into cDNA libraries following the manufacturer’s instructions. Library quality was evaluated by Qubit fluorometry and fragment-size profiling using an Agilent Bioanalyzer or TapeStation. Paired-end 150 bp sequencing was performed on the Illumina NovaSeq 6,000, targeting approximately 30 million read pairs per sample. Raw reads underwent quality assessment with FastQC and adapter/quality trimming with *fastp*. Libraries failing QC requirements were excluded from downstream analyses.

#### Preprocessing, quantification and quality control

Quality-filtered reads were processed with *fastp*, including adapter removal, low-quality base trimming, and filtering of short fragments. Transcript quantification was performed using Salmon v1.10.1 in quasi-mapping mode against the GRCh38 reference transcriptome and summarized to gene-level counts using tximport.

In the blood whole cohort, sequencing depth averaged ∼104 million 151-bp paired-end reads, with GC content between 49% and 55%. Most libraries exhibited duplication levels consistent with poly(A)-selected RNA. Two samples (Schaefer and Morgan, 2011; Nicholson and Suresh Kumar, 2011) failed RNA-seq quality control and were excluded from downstream analyses based on concordant deviations across multiple technical metrics.

Sample 6 showed markedly elevated duplication rates (86.8%), reduced library complexity, and the lowest number of detected genes in the cohort, while sample 5 exhibited borderline library quality characterized by relatively high duplication rates, and fewer detected genes compared with the remaining samples. Importantly, both samples displayed the HAFOUS episignature, indicating that they do not represent a biologically distinct subgroup.

Principal component analysis (PCA) and sample-sample correlation plot (Supplementary Figure S2) reflected these technical differences and were used as supportive diagnostics rather than as exclusion criteria. Complete RNA and RNA-seq quality control metrics are provided in Supplementary Table 2a.

All four fibroblast case and four control libraries met QC criteria. GC content ranged from 47% to 51%, and sequencing depth ranged between 45 and 90 million reads. High duplication levels were observed, as expected for deeply sequenced fibroblast RNA with limited transcriptomic diversity; however, all samples showed sufficient gene detection and coherent clustering and were therefore retained.

Complete QC metrics are provided in Supplementary Tables 2a,b, with graphical summaries in Supplementary Figures S2, S3.

#### Differential gene expression analysis

Gene-level counts were analyzed with *DESeq2* (v1.44) (Love et al., 2014). Transcripts reflecting fewer than 10 total counts across all samples were excluded to improve statistical power. Counts were normalized using the median-of-ratios method implemented in *DESeq2*. For fibroblast analyses, models included age, sex, and age at biopsy as covariates. Differentially expressed genes (DEGs) were defined as those with a Benjamini–Hochberg adjusted *p* < 0.05. For blood analyses, the same pipeline was applied, with models adjusted for age and sex.

#### Functional enrichment and transcription factor analysis

GO Biological Process enrichment was performed separately for blood and fibroblast DEGs using clusterProfiler (Yu, 2024), with gene IDs mapped to Entrez IDs via org. Hs.e.g.,.db. GO with Benjamini–Hochberg adjusted p < 0.05 were considered significant. Gene–concept network plots were generated. Transcription factor (TF) activity was inferred per tissue with decoupleR (Badia et al., 2022) (ULM method) using *DoRothEA* (Garcia-Alonso et al., 2019) regulons, and visualized via volcano plots. The ULM method estimates TF activity by modeling the relationship between target gene expression and TF–target weights derived from curated regulatory networks. DoRothEA provides confidence-weighted TF–target interactions inferred from literature and large-scale datasets. Resulting TF activity scores and pseudo *p*-values were visualized as volcano plots.

#### Expression quantitative trait methylation (eQTM) analysis

For the blood interative sub-cohort, four patients and four controls with high-quality matched DNA methylation and RNA-seq data were included in the eQTM analysis. All four cases and four controls from the fibroblast cohort had matched datasets and were similarly analysed. Cis-eQTM analyses were performed separately for each tissue.

Pearson correlation coefficients were calculated between the mean methylation level (β values) of each DMR and the log-transformed expression of its associated gene within a ±250 kb window (DMR-linked genes). This distance was chosen as it reflects the typical range of gene regulatory interactions (e.g., enhancer) established in large-scale resources such as GTEx and Blueprint Epigenome (Ruiz-Arenas et al., 2022; Küpers et al., 2022). Statistical significance was assessed by comparing observed correlations with a null distribution generated from 1,000 permutations of length-matched random DMRs. Bootstrapped 95% confidence intervals were computed, and associations with p < 0.05 were considered significant. The expression of our genes of interest (significant DEGs/eQTMs), across relevant tissues, including brain, skin, skeletal muscle, liver, and whole blood, were evaluated using GTEx v10 median TPM values.

#### Chromatin context analysis of differentially methylated regions

Differentially methylated regions (DMRs) identified from Blood whole cohort and Fibroblast cohorts mapped to hg38 and converted to hg19 using UCSC LiftOver, retaining only uniquely mapped intervals. Public ChIP-Rx datasets for BCOR, H2AK119ub1, and H3K27me3 from the *USP7*- haploinsufficient neuroblastoma model (GSE263491) were downloaded as bigWig files and imported using rtracklayer. All ranges were harmonized to NCBI chromosome notation. For each replicate, high-signal “peak-like” regions were defined by selecting continuous tiles with ChIP-Rx signal above the 75th percentile; replicates for each mark were merged by concatenation. To test whether DMRs were enriched for Polycomb-associated chromatin features, we generated chromosome- and length-matched background regions using regioneR: randomizeRegions. Background was additionally restricted to CpG islands (UCSC CpGIslandExt) to match CpG density between DMRs and background regions. Average ChIP-Rx signal between DMRs and background were compared using one-sided Wilcoxon rank-sum tests. Overlap was quantified by counting both (i) high-signal tiles overlapping ≥1 DMR and (ii) DMRs overlapping ≥1 tile. Distances between each DMR and the nearest high-signal BCOR, H2AK119ub1, or H3K27me3 region were computed using GenomicRanges: distanceToNearest and log-transformed for visualization. Combinatorial overlap patterns were summarized using UpSet plots. Genomic visualizations were produced using Gviz with EnsDb. Hsapiens. v75 gene annotation alongside DMR and Polycomb tracks.

#### Analysis literature and database review of DMR-Linked genes

To assess the clinical and functional relevance of DMR-linked genes identified in blood and fibroblast cohorts, we performed a literature and database search of 37 candidate genes. For each gene, we searched public databases including OMIM (https://omim.org), GeneCards (https://www.genecards.org) and queried PubMed (September 2025) using gene names and functional aliases in combination with neurodevelopmental and brain-related keywords (i.g., “brain”, “neurodevelopmental disorder”, “brain development”, “cerebral cortex”, “hippocampus”, “prefrontal cortex”) as MeSH terms or in free text [].

Our PubMed search strategy was as follows:

(GENE [tiab] OR “GENE full name” [tiab] OR “GENE gene” [tiab]) AND (“Brain” [Mesh] OR brain [tiab] OR “Neurodevelopmental Disorders” [Mesh] OR neurodevelopment*[tiab] OR “brain development” [tiab] OR “Cerebral Cortex” [Mesh] OR “Hippocampus” [Mesh] OR “Prefrontal Cortex” [Mesh]).

Where appropriate, gene-specific functional keywords or disease associations were added, based on information from OMIM or GeneCards. For each gene, clinical phenotypes and functional roles reported in the literature were documented, and the presence or absence of a known Mendelian phenotype was annotated. The resulting data were summarized in a table for further interpretation.

## Results

We first characterized cohort composition and data quality for both blood and fibroblast samples. We then performed genome-wide DNA methylation analysis followed by transcriptome profiling in each tissue. Finally, we integrated methylation and expression using cis-eQTM analysis and compared regulatory signatures across tissues.

### Blood cohort results

#### Blood cohort characteristics

The blood whole cohort comprised nine patients carrying pathogenic or likely pathogenic *USP7* variants and four healthy controls (Table 1). All nine patients exhibited the characteristic HAFOUS methylation episignature in peripheral blood, including six patients previously reported in our original HAFOUS episignature study. High-quality DNA methylation data were obtained for all participants, forming the blood whole cohort used for differential methylation and DMR analyses.

**TABLE 1:** Blood cohort: molecular characteristics: Overview of blood samples from patients with (likely) pathogenic *USP7*-variants included in DNA methylation and RNA-seq studies. Variant nomenclature follows HGVS guidelines (NM_003470.3; GRCh37/hg19 coordinates).

#	Sex	Age (y)	Cohort	Variant (NM_003470.3)	Variant type	Included in DNA methylation analysis	Included in RNAseq – applied for DEG/eQTM	Reported in earlier study
1	M	9	Blood	c.2132_2140+9del	Indel	✓	✓	✓
2	F	14	Blood	c.1548A>C (p.Leu516Phe)	Missense	✓	✓	—
3	F	20	Blood	c.1988A>C (p.Glu663Ala)/c.2051A>T (p.Asp684Val)	Compound heterozygous missense	✓	✓	✓
4	F	14	Blood	c.713T>G (p.Leu238Arg)	Missense	✓	✓	✓
5	F	8	Blood	arr [GRCh37] 16p13.2 (9036896_9283604)x1	Deletion	✓	NA	—
6	F	9	Blood	c.1357_1355delinsTCCTCCA	Indel	✓	NA	—
7	F	11	Blood	c.1258A>G (p.Lys420Glu)	Missense	✓	—	—
8	M	15	Blood	c.673-1G>C	Splice-site	✓	—	—
9	F	10	Blood	c.2232_2235delGAGA (p.Arg745AsnfsTer5)	Frameshift deletion	✓	—	—
10	F	41	Blood (control)	—	—	✓	✓	—
11	F	36	Blood (control)	—	—	✓	✓	—
12	F	27	Blood (control)	—	—	✓	✓	—
13	F	59	Blood (control)	—	—	✓	✓	—

Abbreviations: SNV, single-nucleotide variant; Indel, insertion/deletion; DEG, differential gene expression; eQTM, expression quantitative trait methylation; –, not applicable. Reported in earlier study* column indicates if the samples were part of the previously published Episignatures of HAFOUS (van der Laan et al., 2023).

RNA sequencing was performed for six out of the nine *USP7* cases together with the same four healthy controls as used for the DNA methylation. Two case samples showed insufficient library complexity and elevated duplication rates and were excluded following RNA-seq quality control. The remaining four *USP7* cases and four healthy controls constituted the blood integrative sub-cohort, which was used for differential gene expression and eQTM analyses.

Comprehensive demographic, molecular, and analytic inclusion details for all samples are provided in Table 1.

#### Genome-wide DNA methylation profiling global hypermethylation

Genome-wide analysis identified 209 significant differentially methylated positions at FDR <0.05, of which 202 (96.7%) were hypermethylated and 7 (3.3%) were hypomethylated. When examining the top 5,000 DMPs (ranked by adjusted P-value), 4,379 (87.5%) were hypermethylated and 621 (12.5%) were hypomethylated, confirming a pronounced global hypermethylation pattern in the cohort. The top 5,000 DMPs ranked by effect size are provided in Supplementary Table 3a.

#### Genome-wide DNA methylation profiling identifies 17 significant DMRs

DMRcate identified 17 significant DMRs (≥5 CpGs, 1 kb window, FDR <0.05), all showing consistent hypermethylation (Supplementary Table 4a). To ensure that methylation patterns were comparable between the full cohort and the integrative sub-cohort, we compared mean β-value distributions for each DMR across both groups. Wilcoxon rank-sum tests showed no meaningful differences between whole-cohort and sub-cohort methylation distributions (all p > 0.05 except one control comparison), with uniformly small effect sizes (Cliff’s delta), confirming that the sub-cohort is representative of the whole cohort (Supplementary Table 4d).

#### Blood transcriptome profiling reveals a limited differential expression signature

After filtering out lowly expressed genes (fewer than 10 total counts across all samples) and retaining only genes expressed in at least 75% of both case and control samples, 15,295 genes remained for differential expression analysis. RNA-seq analysis of whole blood (n = 4 cases; n = 4 controls) identified 33 differentially expressed genes (DEGs) meeting the Benjamini–Hochberg adjusted p < 0.05 significance threshold (Supplementary Table 5a). Only 33 genes were significant and Log_2_ fold changes ranged from approximately −4 to −30, primarily reflecting genes with low or absent expression in one group, as expected given the small cohort size.

Gene set enrichment analysis (GSEA) performed on the ranked blood transcriptome did not identify any gene ontology categories or pathways reaching significance after multiple testing correction. The lack of enrichment is consistent with the limited number of differentially expressed genes and the overall modest transcriptional alterations observed in blood.

#### Integration of DNA methylation and gene expression highlights nine significant eQTM associations

**FIGURE 1:**
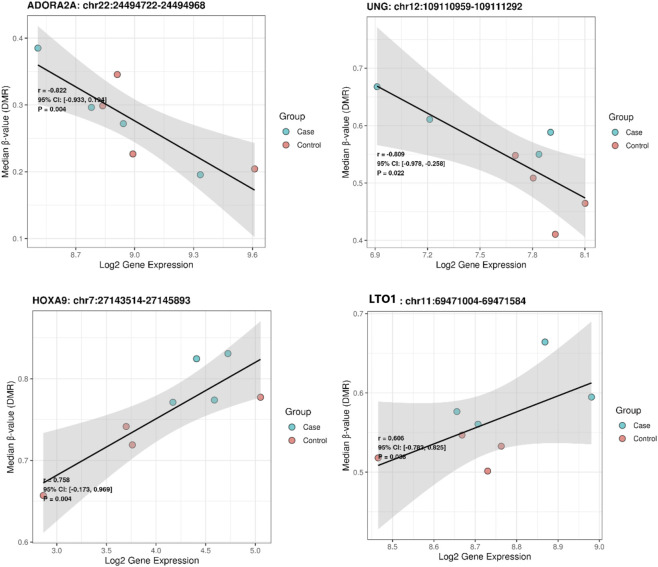
Correlation between DNA methylation and gene expression at selected DMR–gene pairs: Each panel shows a significant DMR–gene association, plotting the median β-value of the DMR against the log_2_-transformed expression of the corresponding gene. Points are color-coded by group (Cases: blue; Controls: red). A linear regression line with its 95% confidence interval (gray shading) is included. Each panel reports the Pearson correlation coefficient (r), its 95% confidence interval, and the P-value, illustrating the strength and significance of the methylation–expression relationship.

Using the 17 blood-derived DMRs, cis-eQTM analysis within a ±250 kb window identified nine significant DMR–gene associations (p < 0.05) (Supplementary Table 6a). These pairs showed consistent methylation–expression relationships indicative of local regulatory effects. Four associations demonstrated strong correlations (r ≥ 0.7) and are presented in Figure 1, whereas the remaining significant associations with moderate correlations (r < 0.7) are shown in Supplementary Figure S3.

### Fibroblast cohort results

#### Fibroblast cohort characteristics

Primary skin fibroblasts were obtained from four patients with (likely) pathogenic *USP7* variants or deletions and four unaffected controls (Table 2). All samples generated high-quality DNA methylation and RNA-seq data and were included in downstream analyses. The same eight samples therefore formed both the fibroblast cohort for methylation analysis and the fibroblast integrative cohort for transcriptome and eQTM analyses.

**TABLE 2:** Fibroblast cohort: molecular characteristics.

#	Sex	Age at biopsy (y)	Cohort	Variant (NM_003470.3)	Variant type	Included in DNA methylation analysis	Included in RNAseq – applied for DEG/eQTM
1	M	20	Fibroblast	c.1454T>G p. (Val485Gly)	SNV	✓	✓
2	F	12	Fibroblast	arr [GRCh37] 16p13.3p13.2 (5752801_9017367)x1	Deletion	✓	✓
3	F	3	Fibroblast	c.3238G>A, p. (Asp1080Asn)	SNV	✓	✓
4	F	19	Fibroblast	arr [GRCh37] 16p13.2 (8969780_9211161)x1	Deletion	✓	✓
5	M	19	Fibroblast (control)	—	—	✓	✓
6	M	21	Fibroblast (control)	—	—	✓	✓
7	F	55	Fibroblast (control)	—	—	✓	✓
8	M	50	Fibroblast (control)	—	—	✓	✓

Abbreviations: SNV, single-nucleotide variant; DMR, differentially methylated region; eQTM, expression quantitative trait methylation; –, not applicable. Variants are described according to HGVS, nomenclature (NM_003470.3) and referenced to GRCh37/hg19 coordinates.

#### Genome-wide DNA methylation profiling balanced hypo- and hypermethylation

Differential methylation analysis of fibroblast samples identified no DMPs that reached FDR significance after multiple-testing correction, consistent with the small cohort size. Therefore, we proceeded with exploratory interpretation based on nominal *p*-values. The top 5,000 CpG sites ranked by nominal significance are summarized in Supplementary Table 3b. Of these, 2,507 (50.1%) were hypermethylated and 2,493 (49.9%) hypomethylated in *USP7*-deficient fibroblasts compared with controls, indicating an approximately balanced global distribution of methylation changes. These DMPs represent candidate loci for downstream region-based and integrative analyses, including DMR identification and eQTM correlation.

#### Genome-wide DNA methylation profiling identifies 2,143 significant DMRs

Using bumphunter (cutoff = 0.15; 100 bootstraps), we identified 2,143 nominally significant DMRs in fibroblasts, comprising 1,314 (61.3%) hypermethylated and 829 (38.7%) hypomethylated regions. No regions passed FWER correction, which is expected given the cohort size and the bumphunter framework, but the set of nominal DMRs was stable across bootstrap iterations. These DMRs formed the basis for downstream integrative analyses, including eQTMs and chromatin contextualization.

#### Differential gene expression identifies 310 genes dysregulated in patient fibroblasts

To investigate transcriptional alterations associated with HAFOUS, we performed differential gene expression analysis comparing fibroblasts derived from affected patients to healthy controls. We observed a total of 310 genes that were significantly differentially expressed (BH adjusted *p* < 0.05, |log_2_FC| >0), comprising 165 upregulated and 145 downregulated genes in patient samples. The expression profiles of these genes with full results are provided in Supplementary Table 5b.

#### Gene set enrichment analysis in fibroblasts

**FIGURE 2:**
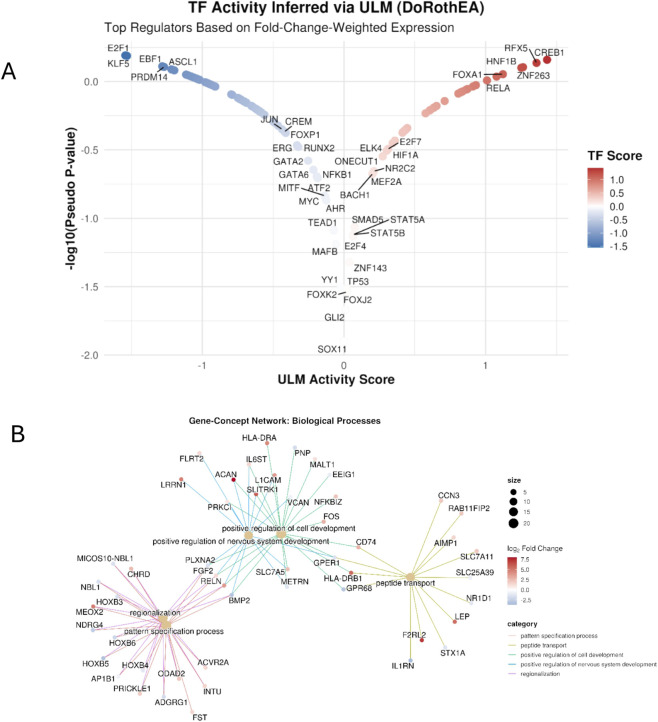
Functional and Regulatory Landscape of Differentially Expressed Genes in *USP7*-associated Fibroblasts. **(A)** Gene–concept network of significantly enriched Gene Ontology (GO) Biological Process terms identified from differentially expressed genes in patient fibroblasts. Each colored node represents a gene, and edges connect genes to enriched GO terms (central nodes). Node colors correspond to log_2_ fold changes, with red indicating upregulation and blue indicating downregulation in patient fibroblasts relative to controls. Prominent processes include *positive regulation of cell development*, *regionalization*, and *peptide transport*, encompassing genes such as *FGF2*, *RELN*, and *BMP2* that contribute to multiple biological categories. The network highlights the coordinated involvement of developmentally regulated genes, including those implicated in nervous system patterning and cellular differentiation. **(B)** Inferred transcription factor (TF) activity in *USP7*-associated fibroblasts. Volcano plot showing TF activity scores estimated using the *decoupleR* ULM method with *DoRothEA* regulons. Colors indicate the direction of inferred TF activity (red, increased; blue, decreased). TF activity reflects changes in the collective expression of each regulator’s target genes rather than in its own transcript levels.

Unbiased gene ontology enrichment analysis of differentially expressed genes in *USP7*-patient fibroblasts revealed significant enrichment in biological processes related to developmental and cellular pathways, notably those associated with nervous system development, including positive regulation of cell development, axon guidance, regionalization, and peptide transport. The gene–concept network visualization (Figure 2A) highlighted key genes contributing to multiple of these processes, such as *FGF2*, *RELN* and *BMP2*.

To investigate upstream regulatory influences, transcription factor activity was inferred using the decoupleR ULM method with DoRothEA regulons. Several TFs showed significantly altered activity scores (Figure 2B), including increased activity of CREB1 and RELA, and decreased activity of ASCL1, E2F1, and KLF5. These activity changes reflect coordinated shifts in target gene expression rather than changes in TF transcript levels.

#### Integrated eQTM analysis reveals coordinated epigenetic and transcriptional dysregulation in *USP7* patient fibroblasts

**FIGURE 3:**
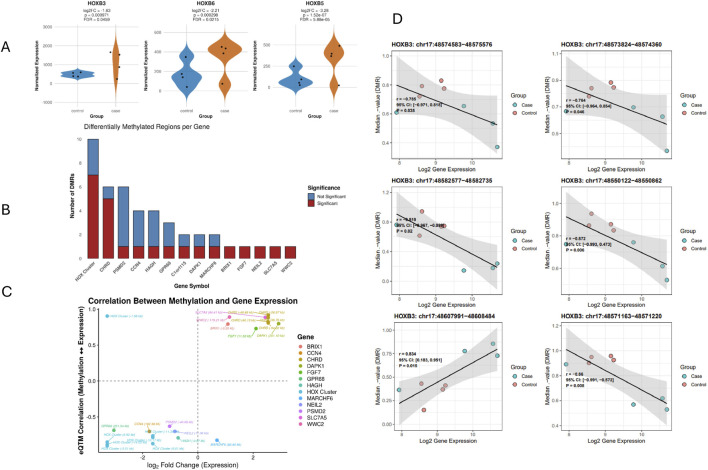
Convergent Differential Expression, Methylation, and eQTM Signatures in the HOXB Cluster **(A)** Violin plots showing downregulation of *HOXB5*, *HOXB6*, and *HOXB3* in patient fibroblasts compared with controls. **(B)** Bar plot showing the number of DMRs per gene; significant DMRs are indicated in red. **(C)** Scatter plot illustrating global relationships between DMR–gene pairs (eQTMs), plotting log_2_ fold change (expression) versus eQTM correlation across 16 overlapping genes. **(D)** Representative examples of DMR–expression correlations for *HOXB3*. Each point represents one sample; black lines indicate regression fits with 95% confidence intervals.

As stated earlier, we identified 2,143 differentially methylated regions (DMRs) between patient fibroblasts and controls using the *bumphunter* algorithm (Δβ cutoff = 0.15; 100 bootstrap iterations). To investigate whether *USP7* haploinsufficiency disrupts transcription through epigenetic mechanisms, we performed an integrative expression quantitative trait methylation (eQTM) analysis, linking DMRs to gene expression within a ±250 kb cis-window. This analysis uncovered 559 significant eQTMs (Supplementary Table 6b). Gene set enrichment of associated loci revealed Biological processes related to developmental and morphogenetic processes, particularly anterior–posterior patterning and skeletal system development, driven largely by HOX gene clusters and embryonic developmental regulators (Supplementary Table 7a). Among these, 16 genes were also differentially expressed, pinpointing candidates under transcriptional and epigenetic regulation (Supplementary Table 7b). Expression analysis confirmed marked downregulation of *HOXB3*, *HOXB5*, and *HOXB6* in patient fibroblasts compared with controls (Figure 3A). Quantification of DMRs per gene revealed that the *HOX* cluster, together with *CHRD*, carried the highest DMR burden, underscoring their potential role as regulatory hotspots (Figure 3B).

A global integration plot demonstrated two distinct regulatory patterns among the 16 overlapping eQTMs (Figure 3C):

- *HOX* cluster genes (*HOXB3*, *HOXB5*, *HOXB6*) showed strong negative correlations (*r* < −0.7, log_2_FC < −2), consistent with hypermethylation-associated repression.
- Conversely, *CHRD*, *DAPK1*, *SLC7A5*, and *WWC2* exhibited positive correlations (*r* > 0.6) with elevated expression, suggesting activation through hypomethylation or permissive chromatin states.

Within the *HOXB* cluster, *HOXB3* is shown as an example. Six DMRs within ±250 kb of *HOXB3* were significantly correlated with expression, including five strong negative correlations (*r* = −0.76 to −0.87, *p* < 0.05) consistent with hypermethylation-mediated silencing, and one positive correlation (*r* = 0.83, *p* = 0.015) (Figure 3D). All significant DMR–gene associations and their corresponding correlation statistics are provided in Supplementary Table 6b, and patient DMR–expression correlation plots are presented in Supplementary Figures S5, S6 for visual assessment of association directionality and strength. Baseline tissue-expression profiles from the GTEx database showed that several genes dysregulated in *USP7*-haploinsufficient fibroblasts including *CHRD*, *SLC7A5*, and *PSMD2* display moderate to high expression levels in skin and multiple brain tissues (Supplementary Figure S7), supporting their potential relevance to both peripheral and neurodevelopmental features of *USP7*-associated syndromes.

#### Cross-tissue concordance of epigenetic and transcriptomic signatures in *USP7* deficiency

**FIGURE 4:**
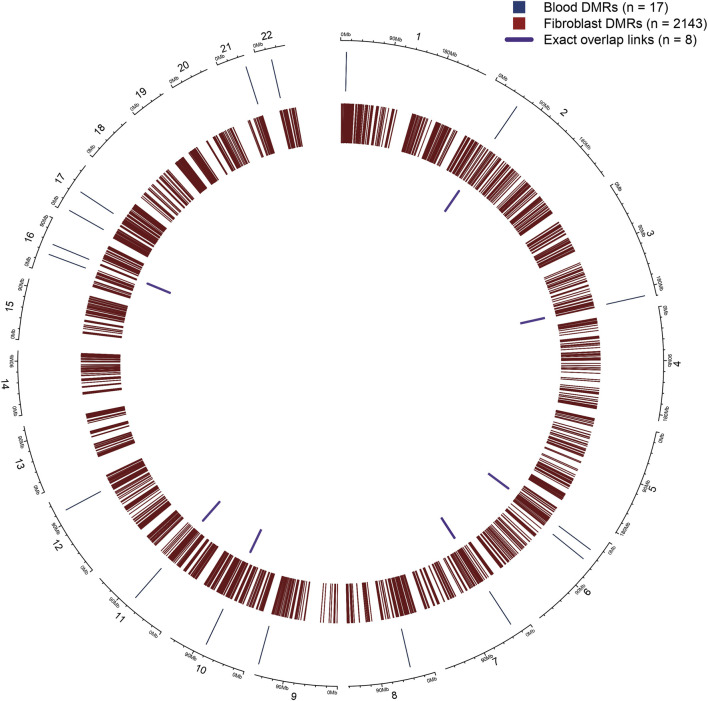
> Circos plot of *USP7*-associated differentially methylated regions (DMRs) in blood and fibroblasts. The outer blue track represents DMRs identified in blood samples, while the inner red track shows DMRs identified in fibroblast samples. Purple ribbons connect loci where DMRs are shared between blood and fibroblasts, indicating exact overlap (≥1 bp) between the two datasets. The number of DMRs in each dataset and the number of overlapping regions are indicated in the legend. This visualization highlights genomic regions where *USP7*-associated epigenetic alterations are consistent across tissue types, pointing to potential stable disease-associated signatures.

To assess whether *USP7*-associated methylation changes are conserved across tissues, we compared fibroblast DMRs (bumphunter; n = 2,143) with blood-derived DMRs identified by DMRcate (n = 17). This analysis identified eight exact overlapping DMRs, located on chromosomes 2, 3, 6, 7, 10, 11, and 16 (Supplementary Table 7c). No additional matches were detected when expanding the window by ±250 bp (Figure 4).

At the gene level, annotation of DMRs within a ±250 kb window revealed five shared genes: *NKAPL, GPR75/ASB3, LRRC15, ZNF423,* and *LRMDA*. These reflect cases where distinct DMRs in each tissue map near the same gene, rather than representing shared genomic regions.

Integration with expression demonstrated limited cross-tissue concordance. Fibroblasts yielded 559 significant eQTMs, whereas blood yielded nine, and only two genes (*HOXA9* and *CNTD1*) were common to both datasets. No differentially expressed genes were shared between tissues.

Together, these findings indicate that only a small fraction of *USP7*-associated methylation changes are conserved across blood and fibroblasts, and that methylation–expression coupling appears largely tissue-specific.

#### DMRs preferentially localize to polycomb-associated chromatin domains

To assess whether differential methylation in *USP7*-haploinsufficient fibroblasts preferentially arises within Polycomb-enriched chromatin, we intersected all 2,143 DMRs with BCOR, H2AK119ub1, and H3K27me3 ChIP-Rx profiles generated in a *USP7*- haploinsufficient neuroblastoma model (Wolf van der Meer et al., 2025). For both hyper- and hypomethylated DMRs, ChIP-Rx signal was significantly higher than matched, chromosome, length-controlled and cpg background regions, indicating robust enrichment of Polycomb-associated features (Wilcoxon rank-sum tests; Supplementary Table 8a). Among hypermethylated DMRs, enrichment was strongest for H2AK119ub1 (W = 1,275,865.5; p = 3.6 × 10^−100^) and BCOR (W = 1,046,247; p = 2.6 × 10^−21^), with H3K27me3 showing a weaker but still significant increase (W = 960,827; p = 2.7 × 10^−7^). Hypomethylated DMRs exhibited similar patterns (H2AK119ub1: W = 521,745; BCOR: W = 432,786; H3K27me3: W = 378,845; all p < 10^−3^).

**FIGURE 5:**
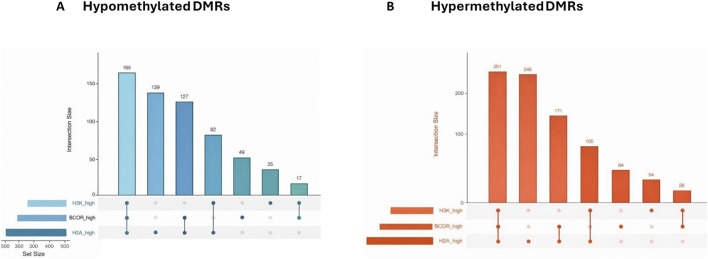
Combinatorial overlap of hypomethylated DMRs with Polycomb-associated chromatin features. **(A)** UpSet plot showing intersections between hypomethylated DMRs (n = 829) and **(B)** Hypermethylated DMRs (n = 1,314) for BCOR (BCOR_high), H2AK119ub1 (H2A_ high), and H3K27me3 (H3K_high) regions derived from SH-SY5Y ChIP-Rx data. Bars represent the number of DMRs overlapping each patient mark or their combinations.

Combinatorial overlap analysis (Figure 5) further demonstrated that many DMRs co-localized with multiple Polycomb features. Among hypomethylated DMRs (n = 829; Figure 5A), 165 overlapped all three marks, 127 overlapped H3K27me3 + H2AK119ub1, and 139 uniquely overlapped H2AK119ub1. Hypermethylated DMRs (n = 1,314; Figure 5B) showed comparable patterns, including 251 overlapping H2AK119ub1 + BCOR, 246 overlapping H2AK119ub1 + H3K27me3, and 105 intersecting all three marks (Supplementary Tables 8c–e).

Genome-wide quantification supported these observations: 72% of all DMRs overlapped high-signal BCOR, 77% overlapped H2AK119ub1, and 26% overlapped H3K27me3 (Supplementary Table 8b). In contrast, only 0.07%–0.14% of all genome-wide high-signal Polycomb tiles overlapped a DMR, indicating that DMRs occupy a highly restricted subset of Polycomb-rich domains.

**FIGURE 6:**
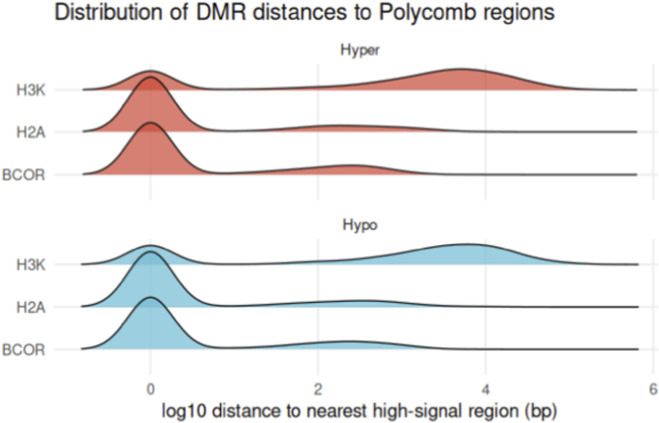
Genomic distance between DMRs and Polycomb-associated chromatin regions. Ridge-density plots showing the log10 genomic distance from each hyper- (top, red) and hypomethylated (bottom, blue) DMR to the nearest high-signal region of BCOR, H2AK119ub1, or H3K27me3 (derived from SH-SY5Y ChIP-Rx data). Both hyper- and hypomethylated DMRs are situated markedly close to PRC1-associated features (BCOR and H2AK119ub1), whereas distances to H3K27me3 are broader, consistent with the more diffuse nature of PRC2 occupancy.

To assess spatial proximity independent of direct overlap, we measured the distance from each DMR to the nearest high-signal Polycomb region. Both hyper- and hypomethylated DMRs were located within tens to hundreds of base pairs from PRC1-associated BCOR and H2AK119ub1 peaks, whereas distances to H3K27me3 were broader (Figure 6), consistent with the diffuse nature of PRC2 occupancy.

Finally, applying the same analysis to the 17 hypermethylated DMRs identified in blood showed a qualitatively similar but more modest pattern. Blood DMRs were also enriched for BCOR and H2AK119ub1 signal relative to background, whereas H3K27me3 showed no significant increase (Supplementary Table 9a). Consistent with fibroblasts, most blood DMRs overlapped PRC1-associated regions (BCOR: 82%; H2AK119ub1: 76%), while a minority overlapped H3K27me3 (18%) (Supplementary Tables 9b–e).

#### Clinical and functional associations of DMR-Linked genes

To contextualize the regulatory loci identified in our methylation and eQTM analyses, we reviewed clinical and functional annotations for 37 genes associated with HAFOUS-linked DMRs using OMIM and the published literature (Table 3). A minority of these genes have established Mendelian phenotypes; examples include *UNG* (immunodeficiency with hyper-IgM type 5), *HAGH* (glyoxalase II deficiency), and *GPR68* (amelogenesis imperfecta). Additional entries such as *MARCHF6*, reported in familial adult myoclonic epilepsy, and *DAPK1*, a kinase involved in neuronal apoptosis and plasticity, further highlight links between the identified loci and known disease or neuronal pathways.

**TABLE 3:** Known OMIM phenotypes or reported functional roles of genes associated with *USP7*-linked DMRs in blood and fibroblasts.

Gene	OMIM #	Blood (top eQTM)	Blood (DEG overlap)	Fibroblasts (top eQTM)	Fibroblasts (DEG overlap)	Reported phenotype/functional role
*UNG*	191,525	✓	–	–	–	Immunodeficiency with hyper IgM, type 5
*HOXA9*	142,956	✓	–	–	–	No specific disease; hematopoietic development
*ADORA2A*	102,776	✓	–	–	–	No Mendelian phenotype; involved in neurological signaling
*LTO1*	607,224	✓	–	–	–	Oral cancer overexpressed protein
*ITGAD*	602,453	✓	–	–	–	Integrin family; no disorder reported
*CNTD1*	618,166	✓	–	–	–	Meiotic cyclin; no disorder reported
*ZKSCAN4*	611,643	✓	–	–	–	Zinc finger protein; no disorder reported
*ZNF165*	600,834	✓	–	–	–	Zinc finger protein; no disorder reported
*CCDC183*	615,955	✓	–	–	–	Testis-enriched protein; no disorder reported
*HAGH*	138,760	–	–	✓	✓	Glyoxalase II deficiency
*CHRD*	603,475	–	–	✓	✓	BMP pathway antagonist; forebrain induction
*SLC7A5*	600,757	–	–	✓	✓	Amino acid transporter; linked to autism spectrum phenotypes
*CCN4*	601,518	–	–	✓	✓	WNT pathway modulator; no syndrome known
*HOXB6*	142,957	–	–	✓	✓	Developmental transcription factor; no known Mendelian disorder
*BRIX1*	618,466	–	–	✓	✓	Ribosome biogenesis; no disorder reported
*GPR68*	604,457	–	–	✓	✓	Amelogenesis imperfecta, hypomaturation type, IIA6
*HOXB3*	142,958	–	–	✓	✓	Developmental transcription factor; no known Mendelian disorder
*FGF7*	600,915	–	–	✓	✓	Growth factor; no known syndrome
*MARCHF6*	613,297	–	–	✓	✓	Epilepsy, familial adult myoclonic, 3
*WWC2*	620,110	–	–	✓	✓	Hippo pathway regulator; neural progenitor control
*NEIL2*	608,933	–	–	✓	✓	DNA glycosylase; no disorder reported
*C1orf115*	–	–	–	✓	✓	Unkown
*PSMD2*	602,840	–	–	✓	✓	Apoptosis regulator; no syndrome
*HOXB5*	​	​	​	✓	✓	Developmental transcription factor; no known Mendelian disorder
*DAPK1*	602,530	–	–	✓	✓	Overexpressed in oral cancer
*HOXB7*	142,962	–	–	✓	–	Developmental transcription factor; no known Mendelian disorder; overexpressed in cancers
*RFLNB*	615,928	–	–	✓	–	Filamin-like repeat protein; no known disorder reported
*SEMA3F*	601,124	–	–	✓	–	Axon guidance and neural development; no clear ID/neuro disorder
*MARK3*	602,678	–	–	✓	–	Visual impairment and progressive phthisis bulbi
*ZNF703*	617,045	–	–	✓	–	Zinc finger protein; no disorder reported
*RUNX2*	600,211	–	–	✓	–	Metaphyseal dysplasia with maxillary hypoplasia with or without brachydactyly
*HOXA3*	142,954	–	–	✓	–	Developmental transcription factor; hindbrain and pharyngeal arch development; no known Mendelian disorder
*PADI1*	607,934	–	–	✓	–	Peptidyl arginine deiminase; episkin differentiation; no known Mendelian disorder
*GOLGA6A*	610,288	–	–	✓	–	Golgin family protein; golgi apparatus organization; no known disorder
*FOXC2*	602,402	–	–	✓	–	Lymphedema-distichiasis syndrome with renal disease and diabetes mellitus

Genes with established OMIM, phenotypes are annotated with their associated disorders. Genes without a known Mendelian disorder are annotated with their primary functional roles when available. Source: OMIM, and PubMed searches (September 2025).

For most genes, no Mendelian disorder has been described, yet their biological functions point to plausible regulatory relevance. These include early developmental transcription factors (*HOXA9*, *HOXB3*, *HOXB6*, *ZKSCAN4*, *ZNF165*), components of ribosome biogenesis (*BRIX1*, *LTO1*), DNA repair (*NEIL2*), and signaling pathways such as WNT and Hippo (*CCN4*, *WWC2*). Additional genes with roles in neurodevelopmental processes such as *SLC7A5*, an amino acid transporter linked to autism spectrum traits, and *CHRD*, a BMP antagonist essential for early forebrain induction provide further functional context. Among these, *MARCHF6* stands out given its involvement in ubiquitination pathways that intersect conceptually with *USP7* function, while the presence of multiple HOX genes (*HOXB3*, *HOXB5*, *HOXB6*) aligns with the broader domain-level regulatory disruptions identified in fibroblasts.

## Discussion

This study provides an analysis of DNA methylation, gene expression, and methylation–expression integration in whole blood and fibroblasts from patients with HAFOUS. These complementary tissues reveal different aspects of *USP7* biology: blood captures the established diagnostic episignature, whereas fibroblasts uncover a broader and more internally consistent regulatory signature. Together, these findings refine the molecular consequences of *USP7* haploinsufficiency in HAFOUS and highlight the relevance of tissue context for detecting chromatin-linked regulatory changes.

A prominent result of this work is the marked difference in regulatory signal strength between blood and fibroblasts. Blood reproduced the previously reported *USP7* episignature but showed limited downstream regulatory effects, with only modest numbers of DMRs, DEGs, and eQTMs. This attenuation is consistent with the strong influence of heterogeneity on peripheral blood methylation profiles (Houseman et al., 2012; Reinius et al., 2012), which can obscure cell-intrinsic regulatory consequences of rare germline variants. In contrast, the homogeneous and lineage-stable nature of fibroblasts enabled the detection of a broader landscape of methylation variation, together with a more substantial transcriptional signature and over 500 significant cis-eQTMs. Similar observations have been made in other chromatin-related neurodevelopmental disorders, where fibroblasts have been used to study disease-associated regulatory alterations than blood (Fahrner and Bjornsson, 2014; Choufani et al., 2015). Within-group analyses of cases and controls in both the blood and fibroblast cohorts revealed directionally consistent methylation–expression relationships across several loci, although statistical power was limited due to small sample sizes. Within-group correlations for the top eQTMs are shown for blood (Supplementary Table S1) and fibroblasts (Supplementary Table S1). Importantly, the preservation of correlation directionality within groups suggests that these eQTMs are not solely driven by case–control differences, but instead reflect underlying regulatory relationships that warrant validation in larger, independent cohorts. Although our fibroblast cohort remains small, the coherence of methylation, expression, and eQTM findings suggests that larger fibroblast datasets would further refine and confirm the regulatory framework associated with *USP7* haploinsufficiency.

Across fibroblast datasets, the most coherent locus of disruption was the HOXB cluster. Several HOXB genes (*HOXB3*, *HOXB5*, *HOXB6*) showed reduced expression, multiple nearby DMRs, and strong negative methylation–expression correlations. Most associated DMRs were located several kilobases from transcription start sites, indicating that the observed regulatory changes likely reflect alterations within broader chromatin domains rather than promoter-specific effects. This configuration is consistent with the known chromatin architecture of HOX loci, which are regulated through extended Polycomb-associated domains rather than isolated promoter elements (Mazzoni et al., 2011; Noordermeer et al., 2011). Additional convergent signals at *CHRD*, *SLC7A5*, *FGF7*, *DAPK1*, and *WWC2* further implicate pathways involved in developmental specification, signaling, and chromatin-linked regulation. Although fibroblasts are not neural cells, the internal consistency across differential methylation, differential expression, and eQTM results indicate that these loci represent core *USP7*-responsive regions.

The genomic context of fibroblast DMRs provides mechanistic support for this interpretation. Using external ChIP-Rx datasets from *USP7*- haploinsufficient neural models, we observed that fibroblast DMRs preferentially overlapped regions enriched for BCOR and H2AK119ub1—marks associated with non-canonical PRC1.1 activity. These findings are aligned with recent experimental work demonstrating that *USP7* dosage regulates the stability of BCOR–ncPRC1.1 and modulates H2AK119ub1 deposition (Wolf van der Meer et al., 2025). Although our DMRs represent nominal associations and the chromatin maps derive from a different cell type, the observed enrichment suggests that *USP7*-sensitive methylation changes arise within PRC1-associated chromatin domains. Within these domains, fibroblasts exhibited concordant hypermethylation and reduced expression of several developmental regulators, providing a mechanistic context for interpreting the regulatory consequences of *USP7* haploinsufficiency.

Cross-tissue comparison further underscores the tissue specificity of these regulatory effects. Only a small number of DMRs overlapped between blood and fibroblasts, and there was minimal convergence in eQTM or DEG associated genes. This pattern is consistent with large-scale multi-tissue datasets demonstrating that methylation–expression coupling is highly tissue dependent (e.g., GTEx; Blueprint Epigenome) (The Genotype-Tissue Expression GTEx project, 2013; Roy et al., 2022). In HAFOUS, these observations suggest that fibroblasts, rather than blood, provide the appropriate cellular context, i.e., less diffuse, for detecting downstream regulatory consequences of *USP7* deficiency. Importantly, this tissue specificity does not diminish the clinical robustness of the blood episignature, which remains reproducible and diagnostically informative; rather, it highlights the difference between diagnostic methylation markers and mechanistically relevant regulatory signatures.

Annotation of genes associated with convergent methylation and expression changes revealed involvement in developmental transcriptional programs, chromatin organization, signaling pathways, and cellular differentiation. The predominance of genes without defined Mendelian disorders is expected for chromatin-associated syndromes, where pathogenic effects often arise from distributed regulatory network changes rather than single-gene dysfunction. The identification of convergent loci including HOX cluster members, *CHRD*, *SLC7A5*, and *FGF7* provides a refined set of candidate pathways through which *USP7* dosage reduction may contribute to neurodevelopmental phenotypes.

Together, these findings indicate that *USP7* haploinsufficiency affects a restricted set of regulatory loci that show enriched overlap with PRC1-associated chromatin regions defined previously in neural models. Within these genomic contexts, fibroblasts reveal a coherent pattern of methylation and transcriptional disruption that is largely undetectable in blood, reflecting differences in tissue composition and regulatory resolution. This integrative dual-tissue analysis therefore provides a mechanistic framework for interpreting the regulatory consequences of reduced *USP7* dosage and establishes a basis for targeted functional studies in disease-relevant cellular models.

### Strengths and limitations in this study

A strength of this study is that we investigated two distinct HAFOUS patient-derived tissues (blood and fibroblast), all carrying previously confirmed (likely) pathogenic *USP7* gene variants. Studying such *ex-vivo*, rather than *in-vitro,* cellular models is generally considered more informative and more likely to reflect clinical outcomes in HAFOUS patients. However, several limitations should be considered when interpreting these findings. Cohort size was small, reflecting the rarity of HAFOUS, and fibroblast DMRs did not reach FWER significance. Moreover, chromatin annotations relied on external datasets rather than tissue-matched profiling. Blood analyses were by definition limited by cell-type heterogeneity. Nonetheless, complementary analytical approaches including incorporation of covariates, permutation-based region calling, and integrative methylation–expression analysis yielded consistent signals across tissues.

### Implications and future directions

The coordinated methylation and expression changes identified in fibroblasts point to a restricted set of *USP7*-sensitive loci, including HOX cluster genes and regulators such as CHRD, SLC7A5, and FGF7. Future work should determine whether these relationships extend to neural lineages using iPSC-derived progenitors or neurons, where developmental timing and cell fate specification can be directly examined. Defining how reduced *USP7* dosage influences PRC1 occupancy, chromatin accessibility, and transcriptional regulation at these loci will be important for establishing causal mechanisms. Broader integrative analyses across related neurodevelopmental disorders, together with mechanistic studies in cellular and *in vivo* models, will be essential for clarifying downstream pathways and assessing potential therapeutic targets.

## Conclusion

The integrated methylation, expression, and eQTM analyses presented here indicate that *USP7* haploinsufficiency affects a restricted set of regulatory loci, with methylation changes preferentially occurring at sites situated within PRC1-associated chromatin domains. Within these regions, fibroblasts showed coordinated hypermethylation and reduced expression of several developmental regulators, including HOX cluster members and genes such as *CHRD*, *SLC7A5*, and *FGF7*. These alterations were far less apparent in blood, consistent with differences in cellular composition and the capacity of each tissue to capture stable regulatory effects. As the specific set of affected genes may vary across neural cell types, further work in disease-relevant models will be required to define their functional impact. Nonetheless, the present data establish a molecular framework for understanding how *USP7* deficiency may influence developmental gene regulation and provide a basis for future mechanistic studies.

## Data Availability

All analysis scripts used for DNA methylation preprocessing, RNA-seq processing, differential expression, DMR calling, eQTM analysis, integrative workflows, and figure generation are available at https://github.com/Manasa-KP/HAFOUS-integrative-analysis. The datasets presented in this article are not readily available because they contain sensitive personal genetic information and cannot be made publicly available under Dutch and EU privacy regulations (AVG/GDPR). Data can be made available upon reasonable request and following approval of a Data Transfer Agreement and institutional data-protection procedures.
