# Supplementary material for: Integrative epigenetic and transcriptomic profiling of whole blood and fibroblasts in Hao-Fountain syndrome
Source: Front Cell Dev Biol. 2026 Feb 20;14:1782599. doi: 10.3389/fcell.2026.1782599 (PMC12963275; doi:10.3389/fcell.2026.1782599)
Supplement: Supplementary file 5 [file DataSheet1.pdf]

Figure S1

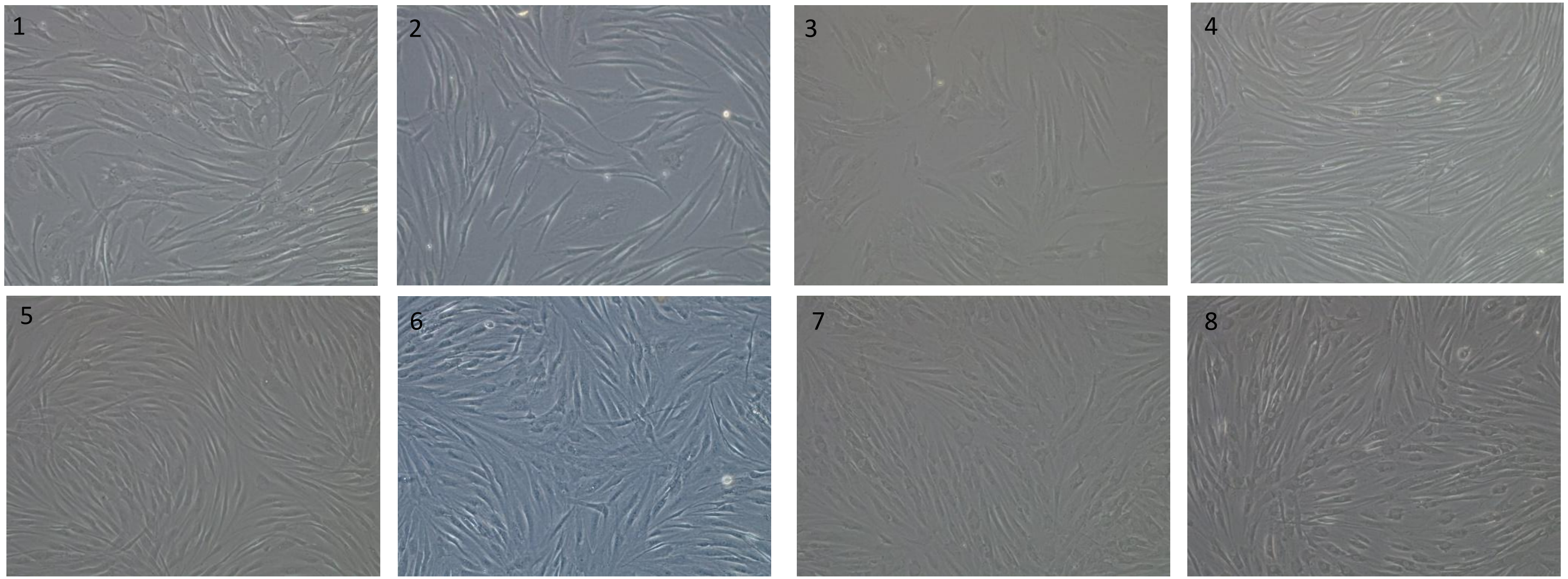

Supplementary Figure S1. Fibroblast Cell culture Images. Representative phase-contrast images of primary dermal fibroblast cultures from all eight study participants (four USP7 cases and four controls). Images illustrate typical fibroblast morphology and confluency prior to DNA and RNA extraction.

Figure S2

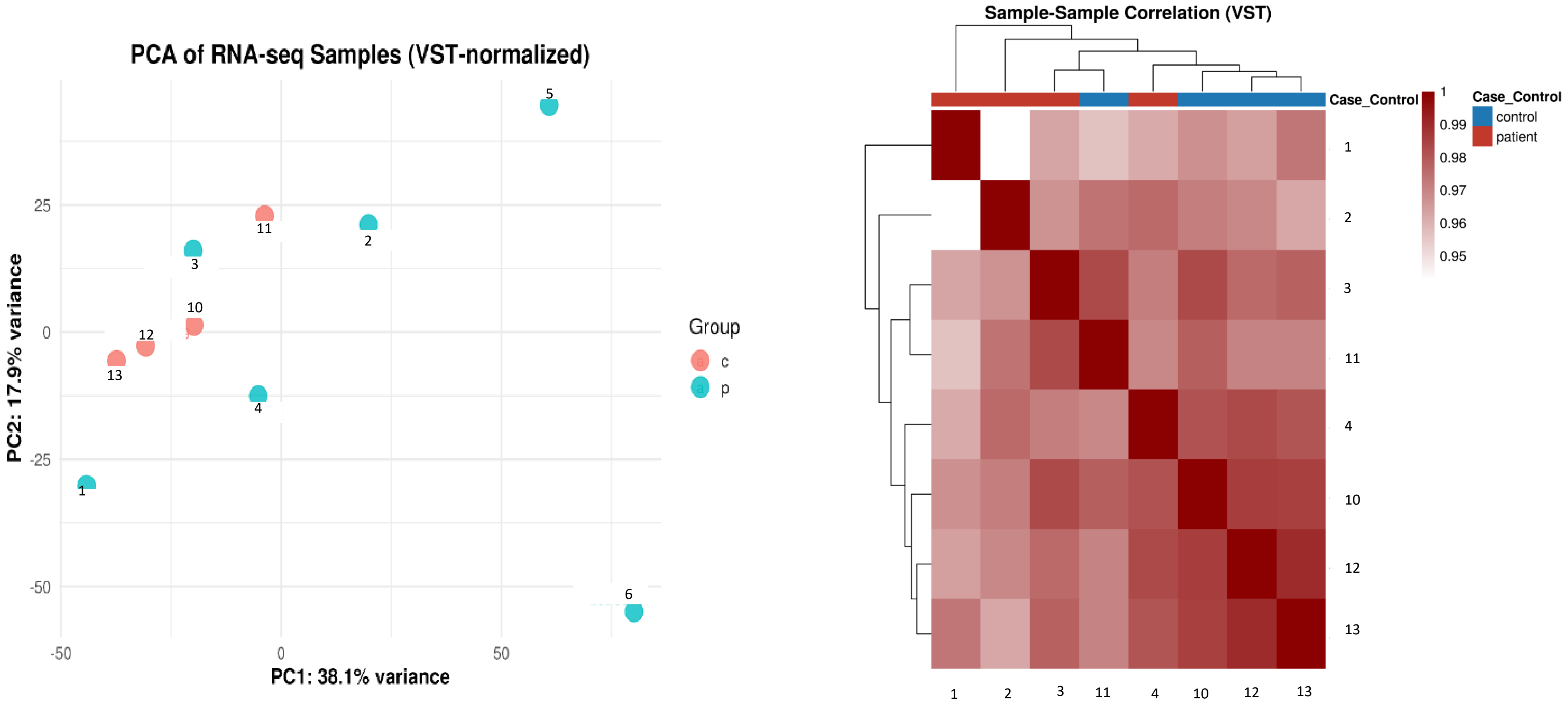

Supplementary Figure S2. Blood RNA-seq quality control.
(A) Principal component analysis (PCA) of variance-stabilized counts showing overall sample structure and identifying two outlier libraries (5 and 6), which were removed prior to downstream analyses.
(B) Hierarchical clustering of sample–sample Pearson correlations (VST-normalized expression), confirming consistent grouping of remaining case and control samples.

Figure S3

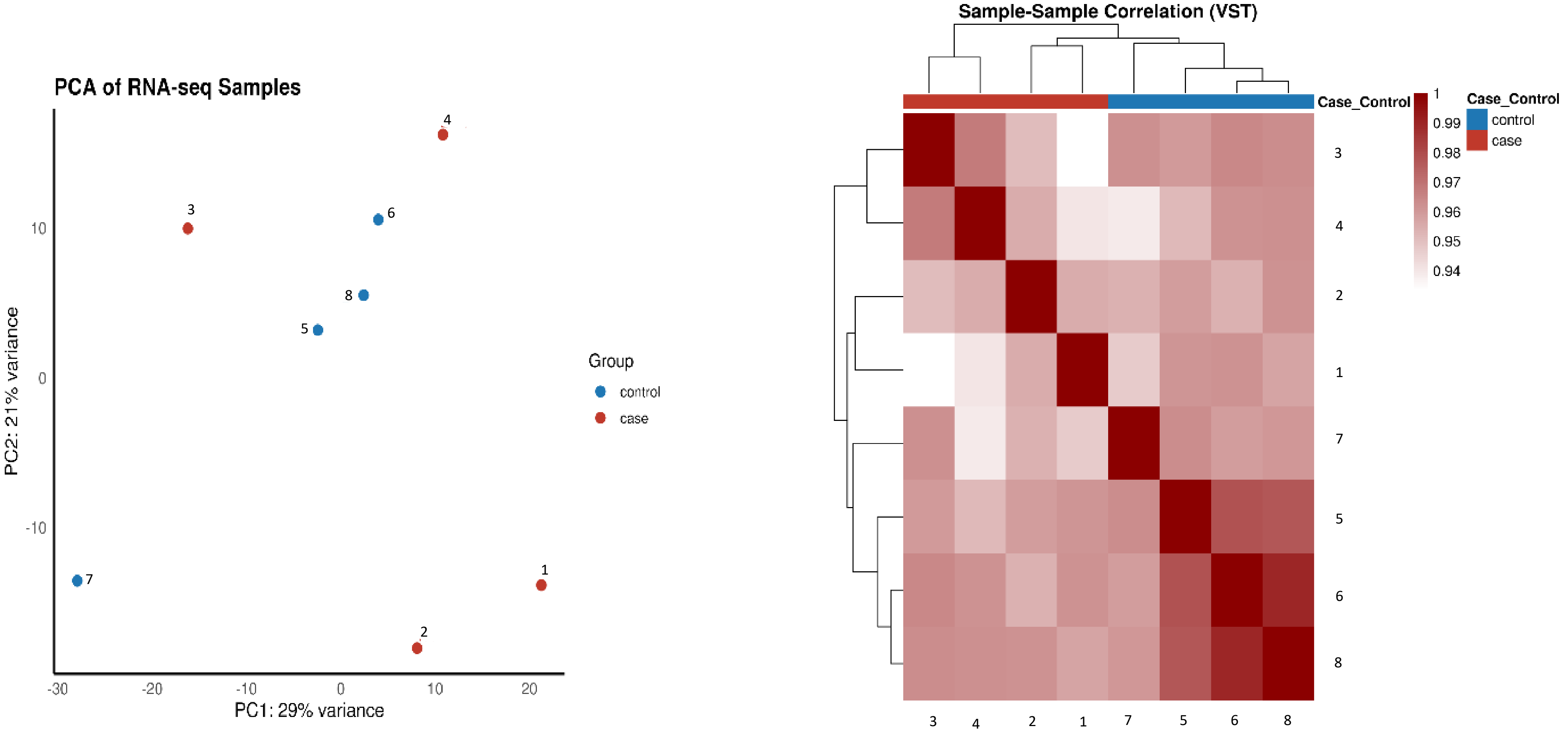

Supplementary Figure S3. Fibroblasts RNA-seq quality control.
(A) Principal component analysis (PCA) of variance-stabilized counts showing overall sample structure.
(B) Hierarchical clustering of sample–sample Pearson correlations (VST-normalized expression), confirming consistent grouping of remaining case and control samples.

Figure S4

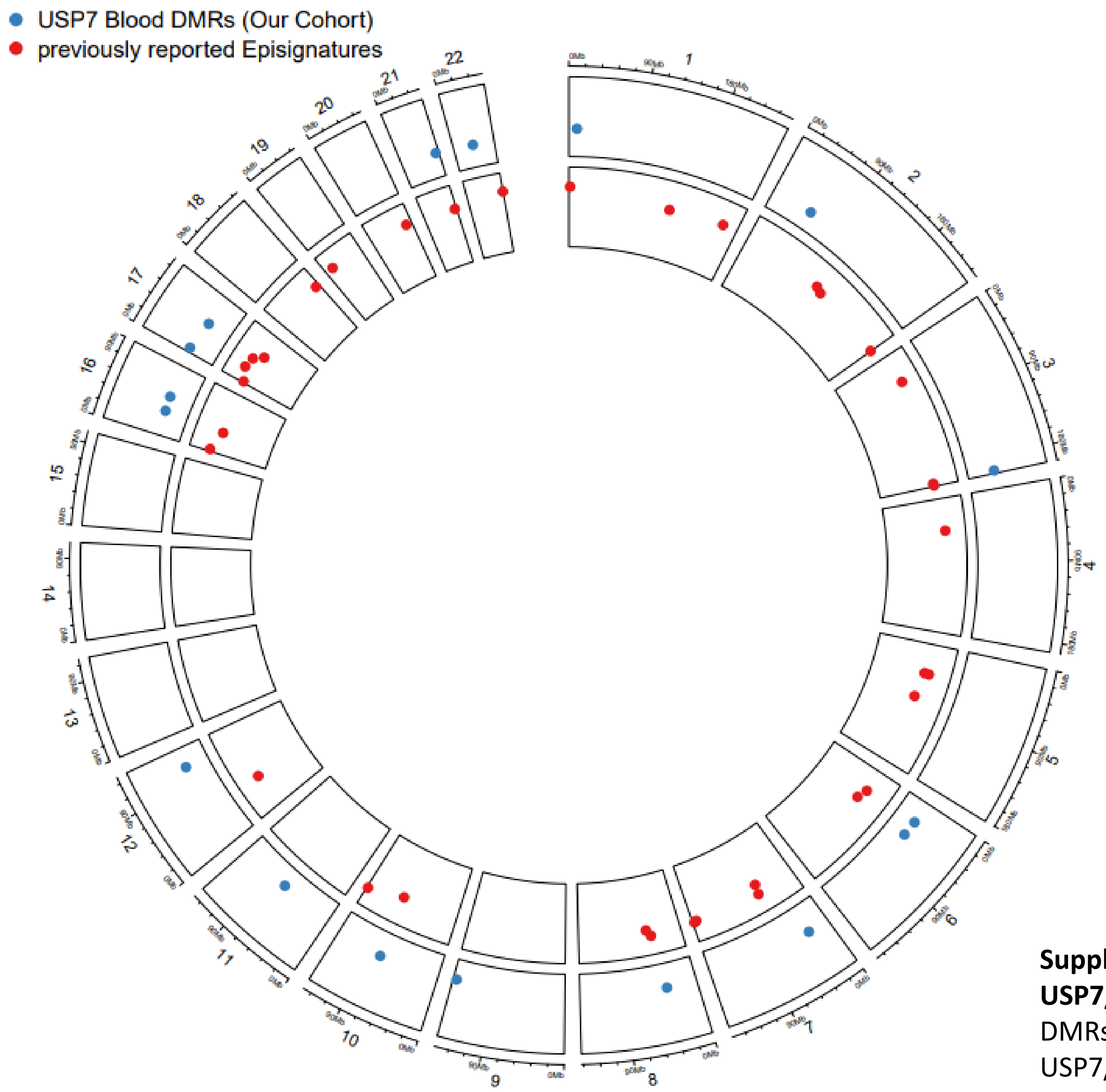

Supplementary Figure S4. Comparison of Blood DMRs With Previously Reported USP7/HAFOUS Episignatures. Circos plot showing the genomic positions of blood-derived DMRs from the current USP7 cohort (blue) compared with DMRs previously reported in the USP7/HAFOUS episignature (red). Each point represents a DMR midpoint plotted across chromosomes (hg19). The visualization illustrates the limited positional overlap between the two datasets. All details are given in Supplementary Table 4e.

Figure S5

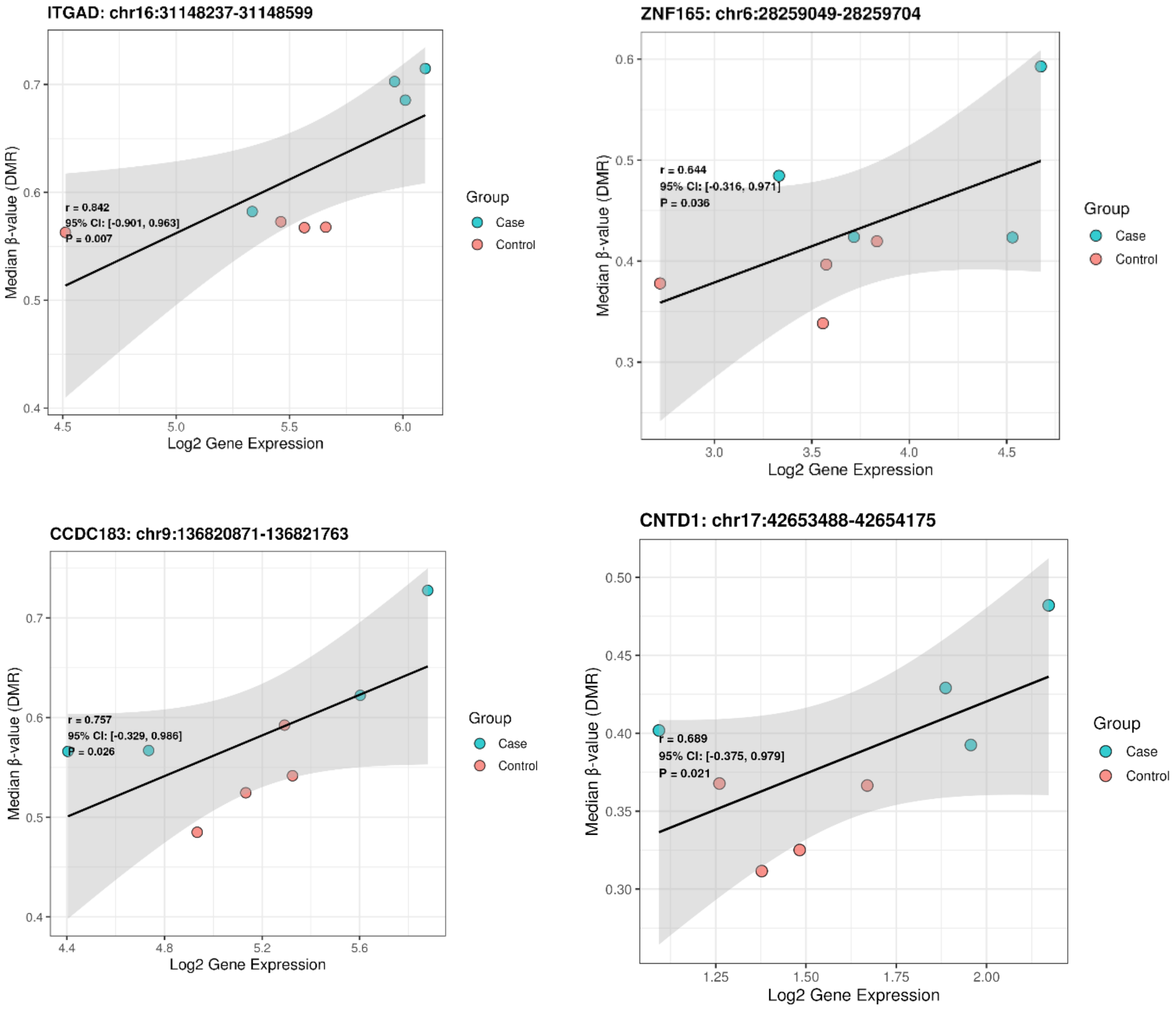

Supplementary Figure S5. Correlation of Differentially Methylated Regions (DMRs) with Gene Expression in Blood cohort. Scatterplots showing the top significant cis-eQTM associations identified in the blood cohort. Each panel displays the correlation between median methylation levels of a DMR and the expression of its linked gene (*ITGAD*, *ZNF165*, *CCDC183*, *CNTD1*). Points represent individual samples, with cases and controls shown separately. Black lines indicate linear regression fits, and shaded areas represent 95% confidence intervals. Reported *r* values, confidence intervals, and *p*-values correspond to Pearson correlations.

Figure S6

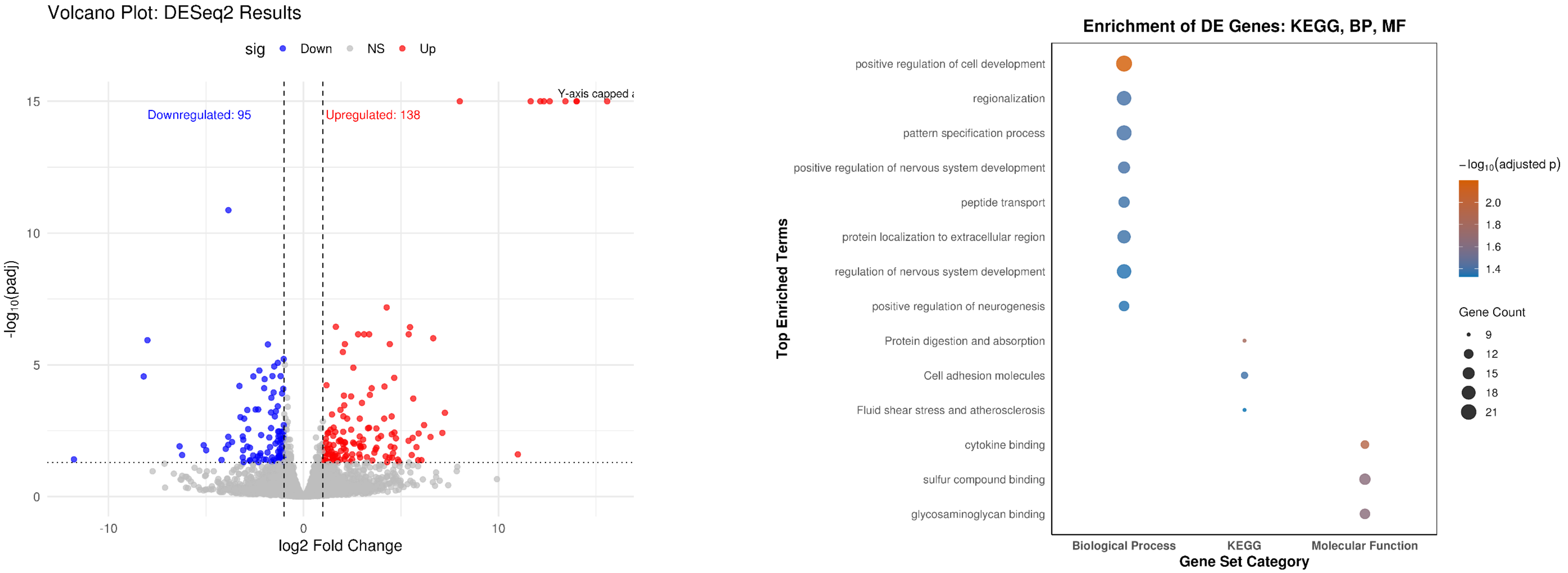

Supplementary Figure S6. Differentially Expressed Genes in the Fibroblast Cohort. (A) Volcano plot of differential gene expression results from DESeq2 comparing fibroblast samples from USP7 cases and controls. Significantly upregulated genes (red) and downregulated genes (blue) are indicated based on Benjamini–Hochberg adjusted $p < 0.05$. (B) Dot plot showing functional enrichment of differentially expressed genes across KEGG pathways, Gene Ontology Biological Processes, and Molecular Function categories. Dot size represents the number of genes contributing to each term, and color denotes $-\log_{10}$(adjusted p) values.

Figure S7

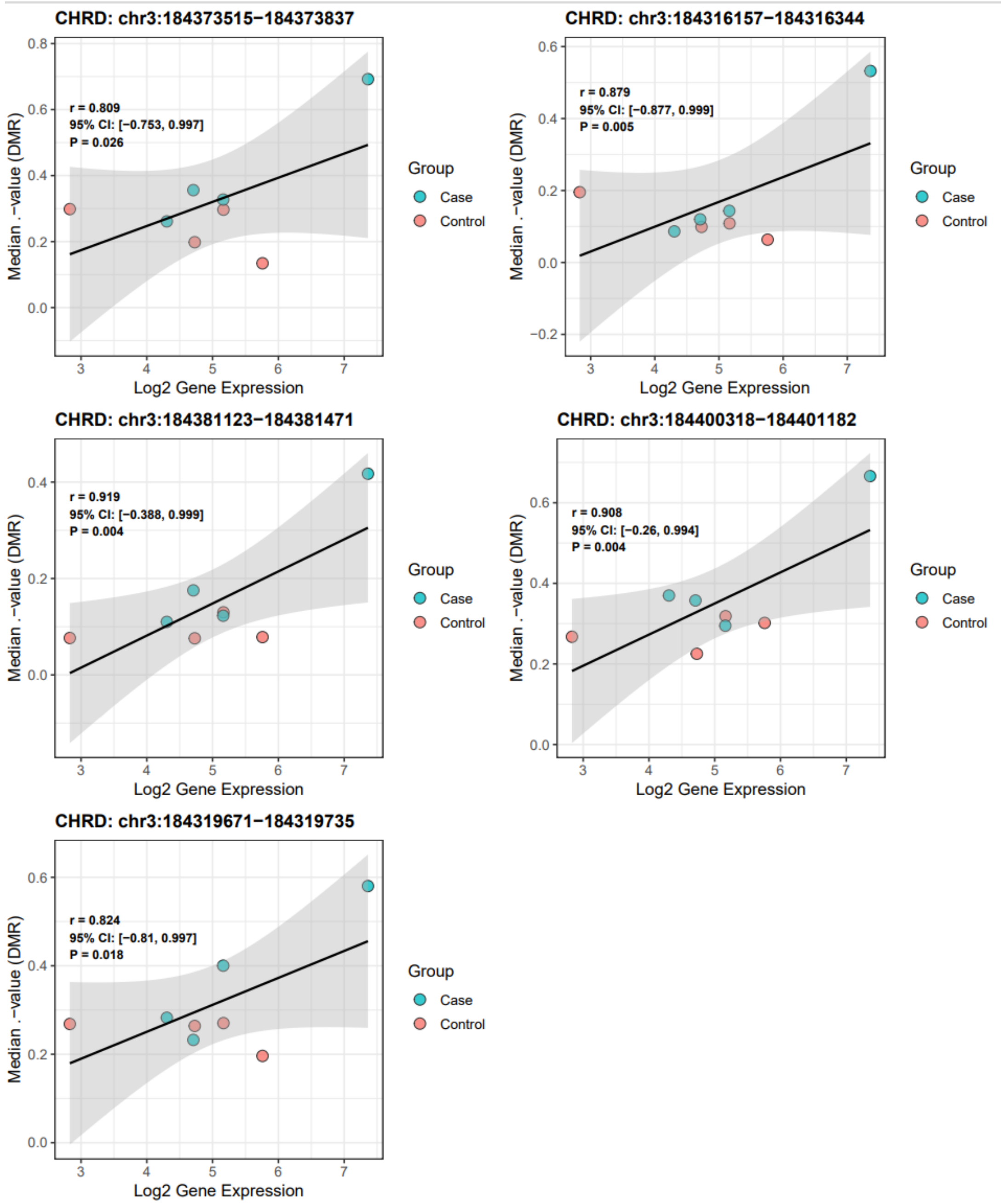

Supplementary Figure S7. Top eQTMs in the Fibroblast Cohort. Scatterplots showing the top significant cis-eQTM associations identified in the fibroblast cohort. Each panel displays the correlation between median methylation levels of a CHRD-associated DMR and **CHRD** gene expression. Points represent individual samples, with cases and controls plotted separately. Black lines indicate linear regression fits, and shaded areas denote 95% confidence intervals. Reported *r* values, confidence intervals, and *p*-values correspond to Pearson correlations.

Figure S8

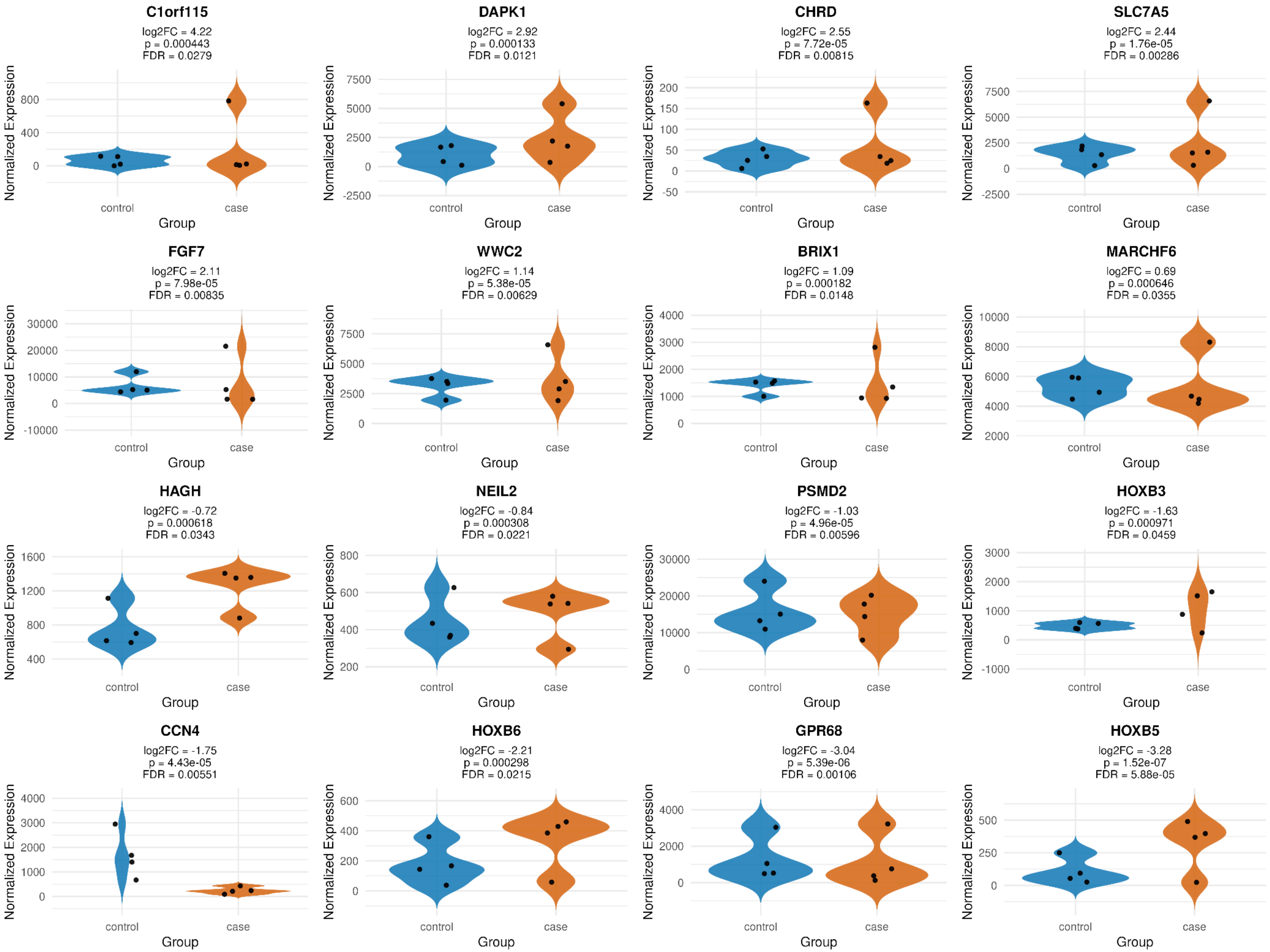

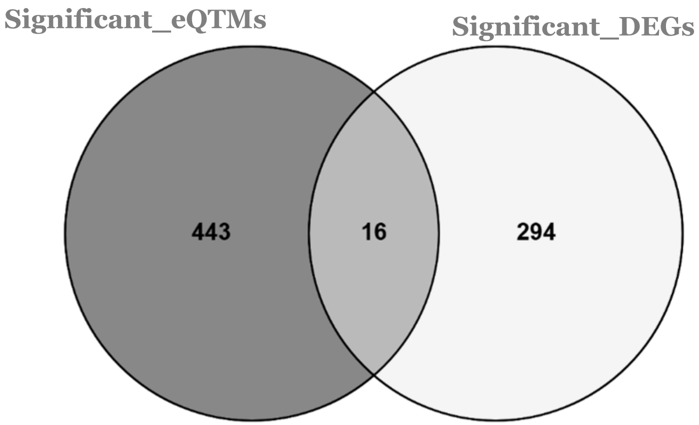

Supplementary Figure S8. Genes Showing Both Differential Expression and Significant eQTM Associations in Fibroblasts
Violin plots show normalized expression levels for the 16 fibroblast genes that were both differentially expressed (DESeq2, FDR < 0.05) and significantly associated with a DMR in cis-eQTM analysis. Each panel displays gene expression in controls and USP7 cases, with $\log_2$ fold change, *p*-value, and FDR reported above. The accompanying Venn diagram summarizes the overlap between all significant cis-eQTMs (n = 443 genes) and significant DEGs (n = 294), with 16 genes shared between the two datasets.

Figure S9

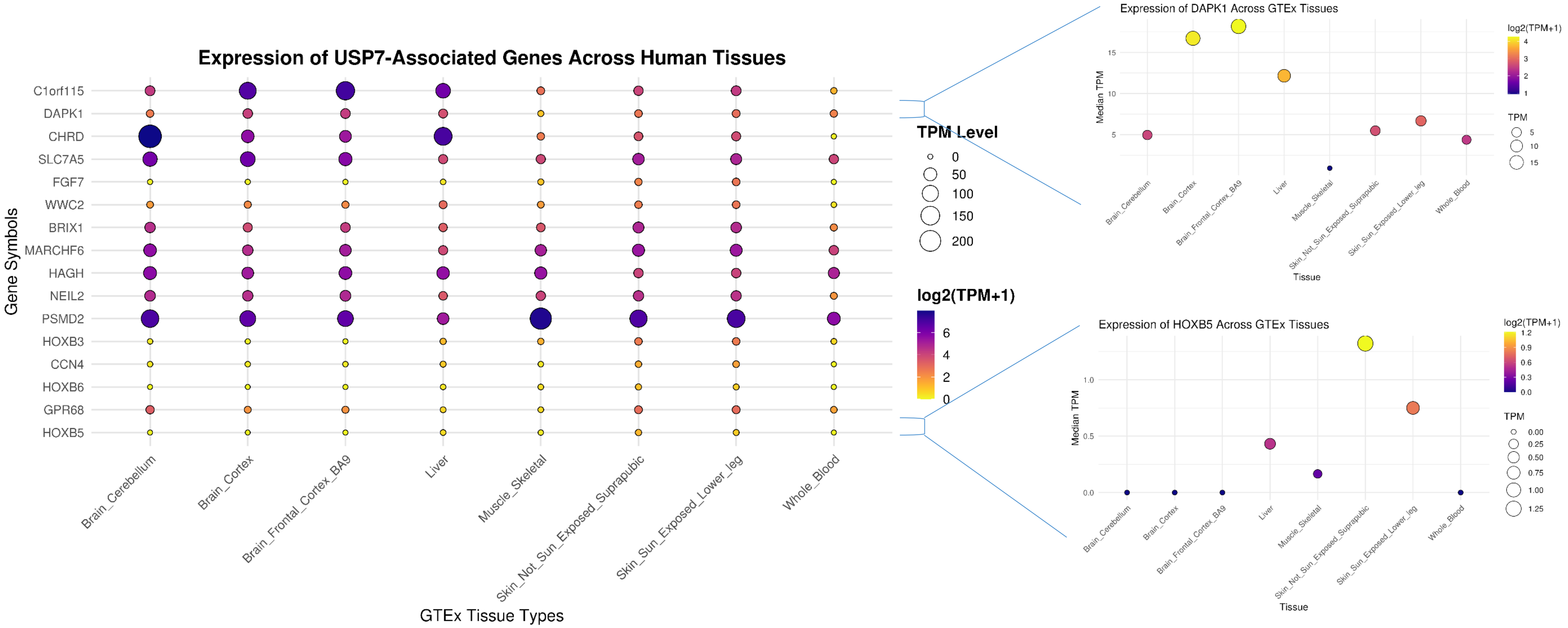

Supplementary Figure S9. Expression of USP7-Associated Genes Across Human Tissues (GTEx).
Dot plots showing median expression of USP7-associated genes across selected GTEx tissue types. In the main panel, dot size reflects TPM values and color corresponds to $\log_2$(TPM+1), illustrating relative expression levels across tissues. Right panels highlight individual examples (*DAPK1* and *HOXB5*) to show gene-specific tissue distribution. These data provide contextual tissue-level expression profiles for genes identified through integrative methylation–expression analyses.
